# Molecular Dynamics Simulations Combined with Markov Model to Explore the Effect of Allosteric Inhibitor Binding on Bromodomain-Containing Protein 4

**DOI:** 10.3390/ijms241310831

**Published:** 2023-06-29

**Authors:** Xiaotang Yang, Yilin Gao, Fuyan Cao, Song Wang

**Affiliations:** 1 Key Laboratory for Molecular Enzymology and Engineering of Ministry of Education, School of Life Science, Jilin University, 2699 Qianjin Street, Changchun 130012, China; yangxt22@mails.jlu.edu.cn (X.Y.); alexandra_gaoyl@163.com (Y.G.); caofy22@mails.jlu.edu.cn (F.C.); 2 The Institute of Theoretical Chemistry, College of Chemistry, Jilin University, 2 Liutiao Road, Changchun 130012, China

**Keywords:** Bromodomain-Containing Protein 4 (BRD4), allosteric regulation, molecular dynamics simulations, Markov model, conformational changes

## Abstract

Bromodomain-Containing Protein 4 (BRD4) can play an important role in gene transcriptional regulation of tumor development and survival by participating in histone modification epigenetic mechanism. Although it has been reported that novel allosteric inhibitors such as ZL0590 have a high affinity with target protein BRD4 and good efficacy, their inhibitory mechanism has not been studied further. The aim of this study was to reveal the inhibition mechanism of allosteric inhibitor ZL0590 on Free-BRD4 and BRD4 binding MS436 (orthosteric inhibitor) by molecular dynamics simulation combined with a Markov model. Our results showed that BRD4-ZL0590 led to α-helices formation of 100–105 compared with Free-BRD4; the combination of MS436 caused residues 30–40 and 95–105 to form α-helices, while the combination of allosteric inhibitors untangled the α-helices formed by the MS436. The results of Markov flux analysis showed that the binding process of inhibitors mainly involved changes in the degree of α-helices at ZA loop. The binding of ZL0590 reduced the distance between ZA loop and BC loop, blocked the conformation at the active site, and inhibited the binding of MS436. After the allosteric inhibitor binding, the MS436 that could normally penetrate into the interior of the pocket was floating on the edge of the active pocket and did not continue to penetrate into the active pocket as expected. In summary, we provide a theoretical basis for the inhibition mechanism of ZL0590 against BRD4, which can be used as a reference for improving the development of drug targets for cancer therapy.

## 1. Introduction

Cancer is still one of the most challenging diseases in this age. The development of cancer is driven and regulated by the accumulation of various changes affecting the structure and function of the genome. The main reason why cancer is difficult to cure is that it involves different kinds of changes at diverse levels in the human body. The gradual accumulation of gene mutation, abnormal transcription, abnormal DNA modification, abnormal regulation of non-coding RNA, abnormal post-translational modification of histones, and other changes will evolve into the possibility of cancer [1,2,3]. Some of these mutations do not involve changes in DNA sequence but can still have a dramatic effect on gene expression, resulting in heritable mutations in gene function, which is known as epigenetics [4,5].

Epigenetics is a regulation of gene expression that affects gene transcriptional activity but does not change DNA sequence [6,7]. Abnormal epigenetic characteristics are involved in the emergence and progression of cancer and may even determine the responsiveness of cancer to treatment [8,9]. Covalent modification of histones is an important mechanism in epigenetic inheritance, and the change in the histone labeling pattern is the main epigenetic modification pathway, which coordinates the progression and metastasis of tumors [10,11,12].

By participating in the epigenetic mechanism of histone modification, the Bromodomain and extra-terminal domain proteins (BET) families play a signaling role in gene expression regulation and epigenetic inheritance [13,14,15]. Therefore, the inhibition of BET families can be used as cancer therapeutic targets. Bromodomain-Containing Protein 4 (BRD4) acts as an epigenetic reader and is a member of the BET protein families of high interest [16,17].

The lead compound ZL0454 [18], discovered by the Department of Pharmacology of the University of Texas Medical Branch (UTMB) in the previous study, has high affinity with the target protein BRD4 and good efficacy. However, it has poor metabolic properties in vivo and low oral bioavailability (e.g., ZL0454 with fairly low C_max_ of 36 ng/mL, AUC_0−t_ of 104 ng·h/mL at the oral dose of 20 mg/kg, and oral bioavailability of 0.5%) [18]. It acts on the classic acetylated lysine KAc binding pocket. The team replaced the N=N structure in ZL0454 with urea, and obtained a new BRD4 inhibitor ZL0590 with improved water solubility and high selectivity (IC_50_ = 90 nM) [19] after systematic structure optimization and a structure–activity relationship study. Culture of the eutectic mixture of ZL0590 and BRD4-BD1 revealed that ZL0590 bound to a new allosteric site [19] rather than the classic acetylated lysine KAc binding pocket for the first time, which was different from common small molecule inhibitors. However, the underlying dynamic mechanism of ZL0590 inhibiting BRD4 has rarely been reported [18].

In this study, molecular dynamics simulation of 500 ns was performed combined with a Markov model to explore the dynamic changes in Free-BRD4, BRD4-ZL0590, BRD4-MS436, and BRD4-MS436-ZL0590. The conformational changes in the binding of BRD4 and inhibitors in the four systems were divided into two groups to analyze: Free-BRD4, BRD4-ZL0590 allosteric inhibitor complex and Free-BRD4, BRD4-MS436 normal inhibitor complex, and BRD4-MS436-ZL0590 double binding complex. Our study provides a theoretical basis for exploring the mechanism of allosteric sites and provides some useful clues for designing inhibitors for cancer treatment.

## 2. Results

### 2.1. Protein Preparation and Structure Stability

**Figure 1:**
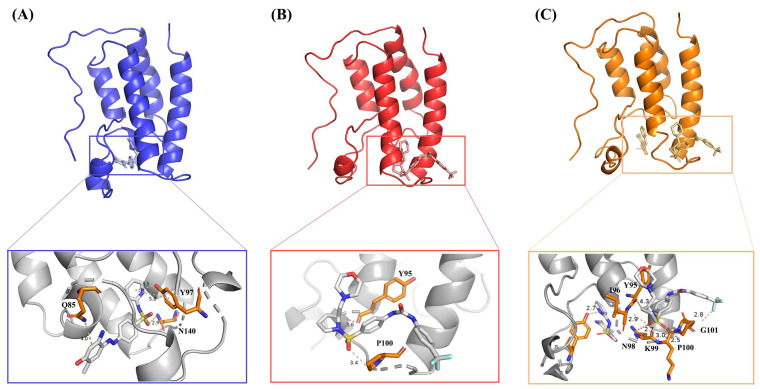
(**A**)The hydrogen bonds between the inhibitor MS436 and BRD4. (**B**) The hydrogen bonds between the allosteric inhibitor ZL0590 and BRD4. (**C**) The hydrogen bonds between the two inhibitors and BRD4.

BRD shares a highly conserved structure consisting of four left-handed bundles of α-helices (αZ, αA, αB, αC) connected by flexible loop regions called ZA loop and BC loop, which are variable in sequence and form acetyl binding sites [20]. Similar in structure to the Bromodomain-Containing family proteins, the BRD4 protein consists of four α-helices (A, B, C, and Z), two loops (ZA and BC), a ZA channel, WPF pocket, and a cavity between four α-helices. BRD4 uses these cavities to mediate histone recognition [20]. Figure 1A–C show the hydrogen bonds between the inhibitor MS436, the allosteric inhibitor ZL0590, the two inhibitors, and BRD4. There were four hydrogen bonds between BRD4 (Q85, Y97, N140) and MS436 and two hydrogen bonds between BRD4 (Y95, P100) and ZL0590. After using molecular docking to bind both inhibitors, there was one hydrogen bond between BRD4 (C96) and MS436 and six hydrogen bonds between BRD4 (Y95, I96, N98, K99, P100, G101) and ZL0590. The more hydrogen bonds there are between complexes, the more stable they are.

**Figure 2:**
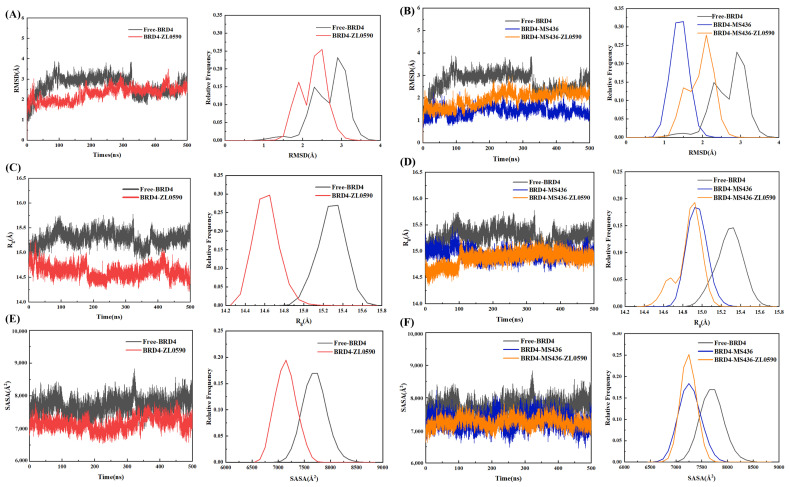
(**A**) The RMSD diagrams of Free-BRD4 and BRD4-ZL0590. (**B**) The RMSD diagrams of Free-BRD4, BRD4-MS436, and BRD4-MS436-ZL0590. (**C**) The R_g_ diagrams of Free-BRD4 and BRD4-ZL0590. (**D**) The R_g_ diagrams of Free-BRD4, BRD4-MS436, and BRD4-MS436-ZL0590. (**E**) The SASA diagrams of Free-BRD4 and BRD4-ZL0590. (**F**) The SASA diagrams of Free-BRD4, BRD4-MS436, and BRD4-MS436-ZL0590.

To evaluate the convergence of each system and ensure the reliability of subsequent sampling strategies, the root mean square deviation (RMSD) of the Cα atom was obtained (Figure 2A,B). The four systems Free-BRD4, BRD4-MS436, BRD4-ZL0590, and BRD4-MS436-ZL0590 reached equilibrium after about 200 ns and remained stable in the simulation process. BRD4-MS436 stabilized at around 1.5 Å, while BRD4-ZL0590 and BRD4-MS436-ZL0590 fluctuated at around 2.5 Å.

The radius of gyration (R_g_) was calculated to reveal the variation in protein tightness in the four systems during simulation. It can be seen from Figure 2C,D that, compared with Free-BRD4, the R_g_ value of BRD4-ZL0590 decreased significantly, indicating that conformation changed after the allosteric inhibitor binding. Compared with BRD4-MS436, the frequency spectrum of the R_g_ value of BRD-MS436-ZL0590 was wider, indicating that the binding of allosteric inhibitors had an effect on the conformation of MS436.

It can be seen from Figure 2E,F that the SASA value of free protein stabilized at about 7500~8500 Å^2^ after 200 ns. Compared with Free-BRD4, the SASA value of BRD4-ZL0590 decreased, and the final value stabilized at about 7000 Å^2^, indicating that the binding of allosteric inhibitors reduced the hydrophilicity of the protein. Compared with BRD4-MS436, the SASA values of BRD-MS436-ZL0590 were concentrated in frequency, indicating that the binding of allosteric inhibitors made the conformation caused by MS436 more hydrophobic.

In summary, after 500 ns MD simulation, all four systems were stable and could be used for further study.

### 2.2. Dynamical Cross-Correlation Matrix Analysis

**Figure 3:**
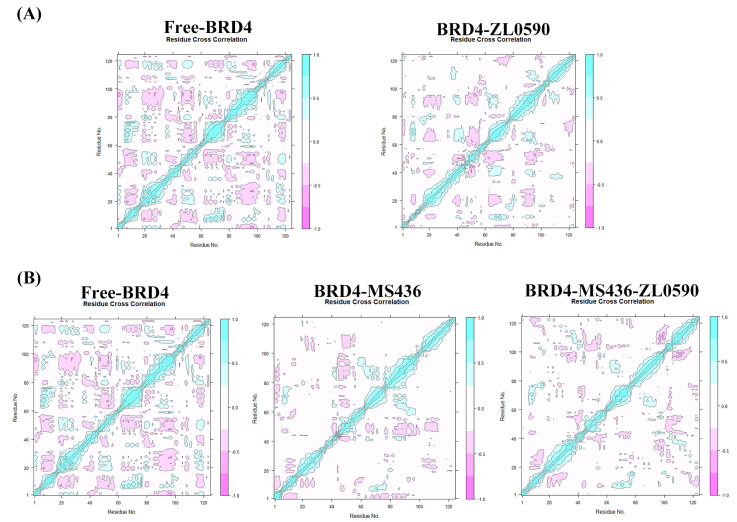
(**A**) The Dynamical Cross-Correlation Matrix diagrams of Free-BRD4 and BRD4-ZL0590. (**B**) The Dynamical Cross-Correlation Matrix diagrams of Free-BRD4, BRD4-MS436, and BRD4-MS436-ZL0590.

Dynamical Cross-Correlation Matrix (DCCM) analyses of all Cα atoms are shown in Figure 3A,B. It can be seen from Figure 3A that the color of the BRD4-ZL0590 system was lighter than that of Free-BRD4 in the correlation matrix diagram, indicating that in the MD simulation process, the BRD4-ZL0590 system had weak flexibility and good stability. As can be seen from Figure 3B, the color of the BRD4-MS436 system was the lightest in the correlation matrix diagram, indicating that the BRD4-MS436 system had the weakest flexibility and the best stability in the MD simulation process.

### 2.3. Flexibility Analysis of BRD4 Protein Active Region

**Figure 4:**
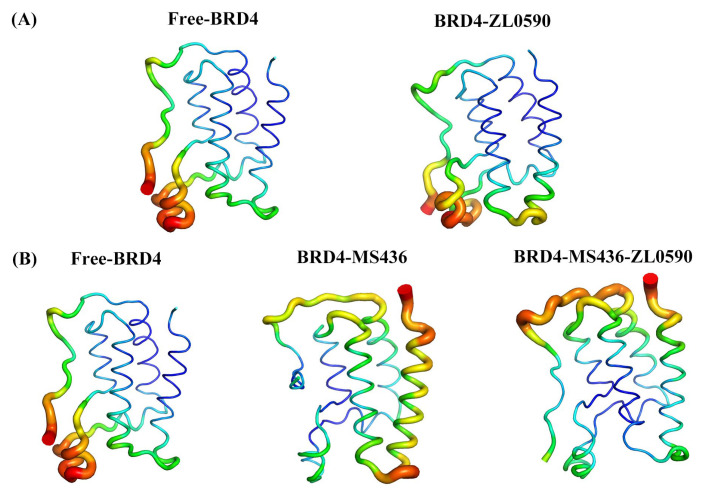
(**A**) The B-factors diagrams of Free-BRD4 and BRD4-ZL0590. (**B**) The B-factors diagrams of Free-BRD4, BRD4-MS436, and BRD4-MS436-ZL0590.

Figure 4A,B show the value of B-factor during MD simulation. B-factor reflects the protein’s flexibility. The higher the B-factor in the protein structure, the better the protein mobility. As can be seen from Figure 4, in the MD process, the binding of MS436 reduced the B-factor near the active site, while the simultaneous binding of MS436 and ZL0590 inhibitors reduced the B-factor of the entire BC loop and ZA loop domain, and the active region of BRD4 experienced the weakest flexibility and the greatest stability.

By using temperature factors to visualize the stability of proteins at different locations, the dynamics and flexibility of different regions of the protein structure can be more intuitively observed [21]. The comparison of the values of the temperature factors of the active sites in the molecular structure of the proteins of the four systems can help us better analyze the differences in the flexibility of each site of the proteins, and further study the relationship between the structure and function of proteins.

**Figure 5:**
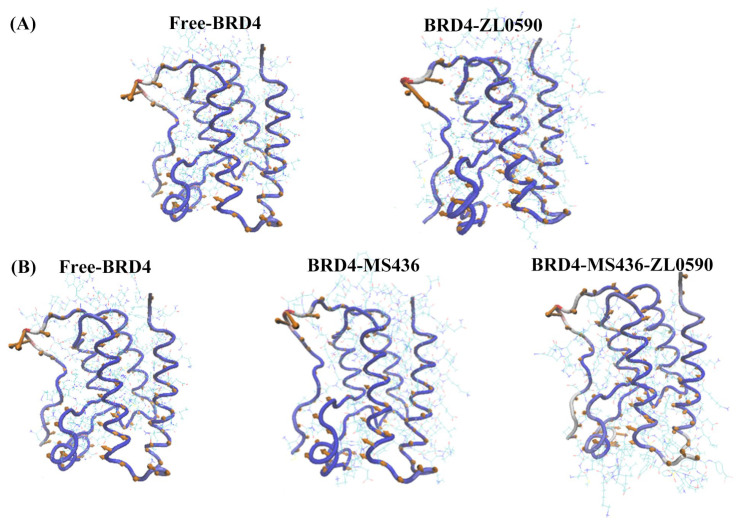
(**A**) The Prody diagrams of Free-BRD4 and BRD4-ZL0590. (**B**) The Prody diagrams of Free-BRD4, BRD4-MS436, and BRD4-MS436-ZL0590.

The Prody arrow plot represents the trend of movement, and the length of the arrow represents the amplitude of movement. It can be seen from Figure 5 that the movement trend of the active regions of the four systems was almost the same, and the motion amplitude was slightly different, among which Free-BRD4 had the largest motion amplitude, BRD4-MS436 had the smallest motion amplitude, and the structure was the most stable. In addition, large fluidity was observed in the 10–16 disordered region of residues.

### 2.4. Conformational Changes during MD Simulations

**Figure 6:**
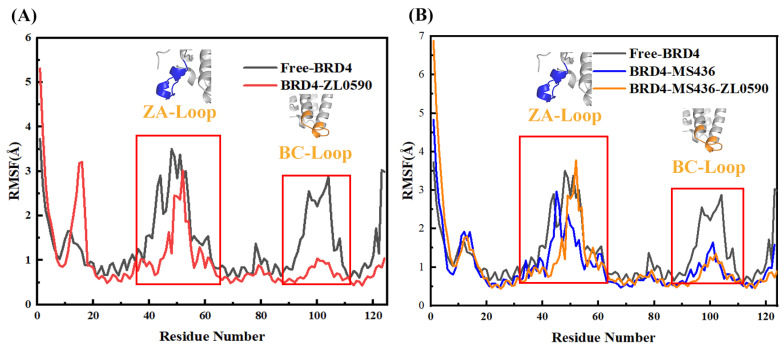
(**A**) The RMSF diagram of Free-BRD4 and BRD4-ZL0590. (**B**) The RMSF diagram of Free-BRD4, BRD4-MS436, and BRD4-MS436-ZL0590.

To compare the effect of ZL0590 on BRD4, root mean square fluctuations (RMSF) of Cα atoms were calculated (Figure 6A,B). In general, all four systems showed similar RMSF distributions with few exceptions. For the four systems, the major changes in RMSF occurred at residues 30–60 and 90–110. Free-BRD4 had greater flexibility than BRD4-ZL0590. At residues 30–60, the flexibility of BRD4-MS436 was less than that of BRD4-MS436-ZL0590. The flexibility of BRD4-MS436 was greater than that of BRD4-MS436-ZL0590 at residues 90–110. The results suggest that binding allosteric inhibitors might enhance the interaction between BRD4 and MS436 between 90 and 110. Residues 90–110 in Free-BRD4 showed high flexibility, while in BRd4-MS436-ZL0590, their RMSF was reduced compared to BRD4-MS436. The results showed that the binding of allosteric inhibitors enhanced the interaction between BRD4 and inhibitors. The above differences in the flexibility of residues 30–60 and 90–110 might affect the structural fluctuation of inhibitors binding to BRD4, leading to different sensitivities of inhibitors to BRD4.

**Figure 7:**
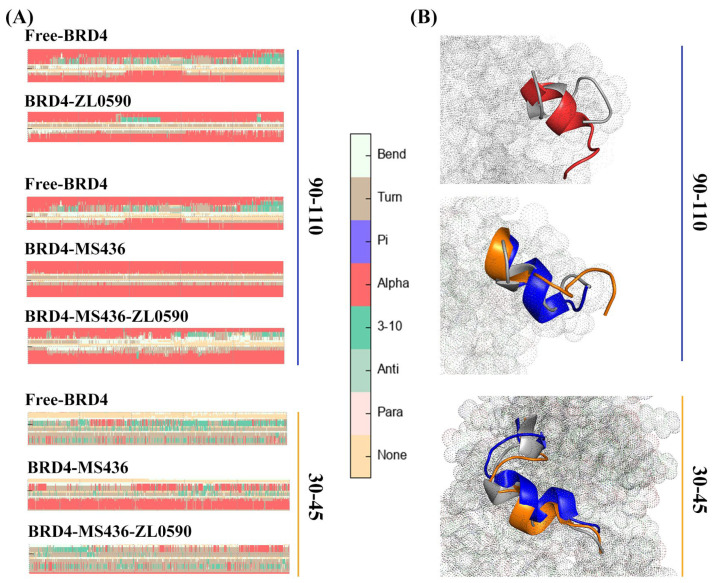
(**A**) The secondary structure changes’ probability of Free-BRD4 and BRD4-ZL0590, BRD4-MS436 and BRD4-MS436-ZL0590 in residues 90–110 and 30–45. (**B**) The 3D structure changes in the four systems.

The study of protein secondary structure is an indispensable part of molecular dynamics simulation. Between the Free-BRD4 and BRD4-ZL0590 systems, the change in the secondary structure of residues 90–110 is shown in Figure 7A. The 3D structure change in residues M90-A110 in conformation is shown in Figure 7B (where gray represents Free-BRD4, red represents BRD4-ZL0590, blue represents BRD4-MS436, and orange represents BRD4-MS436-ZL0590). As can be seen from Figure 7B, α-helices accounted for a large proportion in the 90–110 domain of residues of BRD4-ZL0590, while in Free-BRD4, α-helices hardly existed. Similarly, for Free-BRD4, BRD4-MS436 had the most α-helices, followed by BRD4-MS436-ZL0590. Compared with Free-BRD4, BRD4-MS436 had the most α-helixes in residues 30–45, followed by BRD4-MS436-ZL0590. In general, the secondary structure of BRD4 changed after inhibitor binding. Compared with Free-BRD4, the binding of MS436 caused residues 30–40 and 95–105 to form helices, while the binding of ZL0590 caused the partially uncoiled helices formed by MS436.

**Figure A1:**
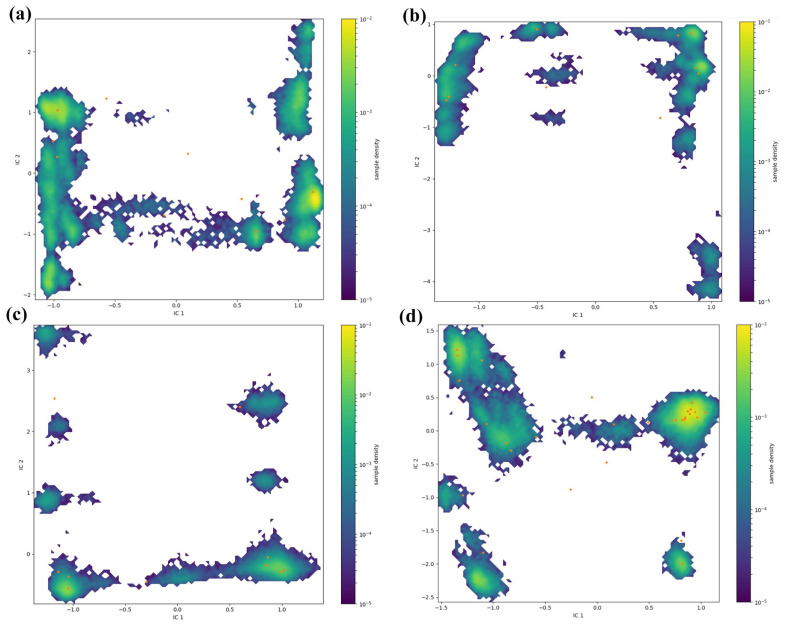
(**a**)The results of K-means diagrams of Free-BRD4. (**b**) The results of K-means diagrams of BRD4-ZL0590. (**c**) The results of K-means diagrams of BRD4-MS436. (**d**) The results of K-means diagrams of BRD4-MS436-ZL0590.

The entire process of Markov model calculation was carried out using the pyemma software package (version number: 2.5.12) [22]. Taking the trajectory of BRD4-MS436 as an example, 10 molecular trajectories were transformed and reduced to 10 two-dimensional arrays with dimensions of 5000 × 139. Among them, 10 two-dimensional arrays corresponded to 10 trajectories, and each trajectory corresponded to a two-dimensional array. The first dimension of the two-dimensional array (5000) represented 5000 frames per trajectory; the second dimension 139 indicated that the angle information used to quantify the residual skeleton in each frame was 139 dimensions. Afterwards, k-means clustering was performed, with a k-value of 10 selected during the clustering process. The clustering results are shown in Figure A1.

**Figure A2:**
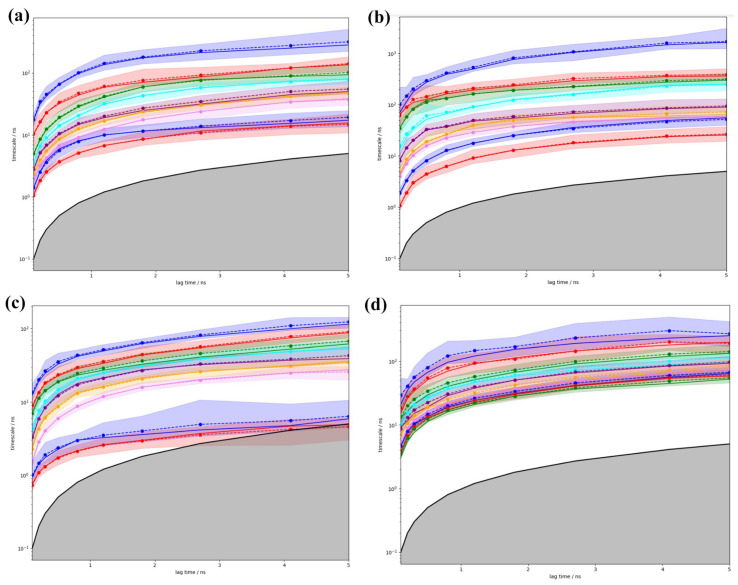
(**a**) The lag time determination of Free-BRD4. (**b**) The lag time determination of BRD4-ZL0590. (**c**) The lag time determination of BRD4-MS436. (**d**) The lag time determination of BRD4-MS436-ZL0590.

After successful clustering, it is necessary to use this clustering result to construct a Markov model. Before constructing a Markov model, the next step is to determine the lag time value. The minimum lag time when the relaxation time scale reaches convergence is the required lag time, which is the Markov time. Observing Figure A2, it can be seen that the relaxation time scale of the entire system tended to converge when the lag time was 1 ns. Therefore, in this experiment, a lag time of 1 ns was chosen. Since the step size used to calculate the molecular simulation trajectory was 0.1 ns, the lag time was 10 steps.

**Figure A3:**
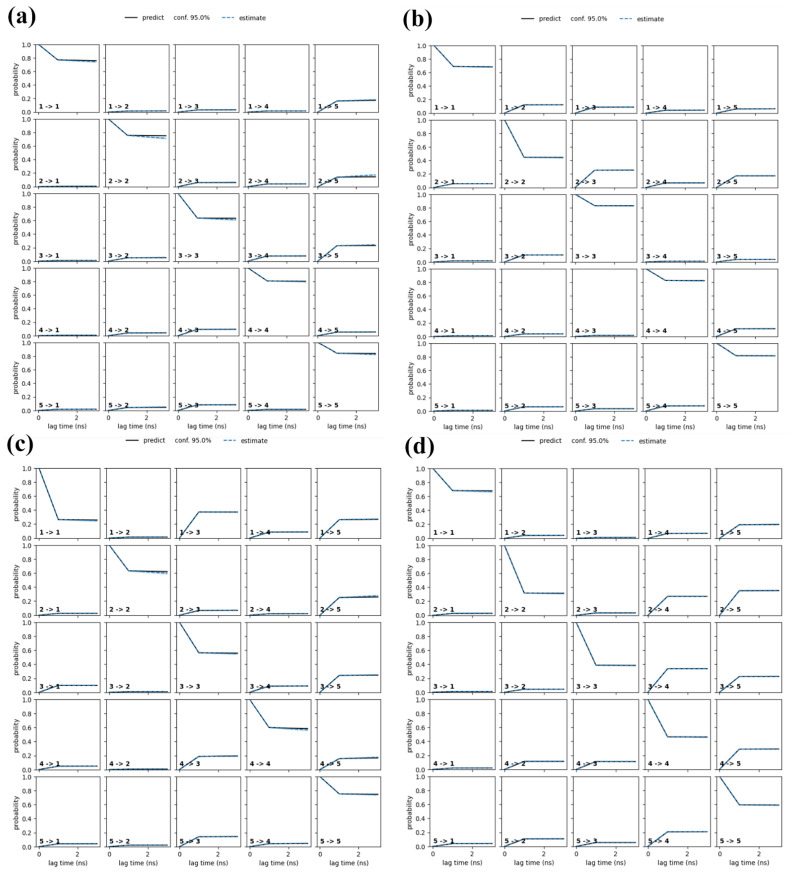
(**a**) The results of ck-test of Free-BRD4. (**b**) The results of ck-test of BRD4-ZL0590. (**c**) The results of ck-test of BRD4-MS436. (**d**) The results of ck-test of BRD4-MS436-ZL0590.

Afterwards, the obtained lag time and clustering results could be used to construct a Markov model and calculate the transition probability matrix. After constructing the Markov model and obtaining the transition probability, this experiment carried out a ck-test to detect the Markov property of the constructed model. In the ck-test algorithm, the k was selected as 5, which meant that the number of macroscopic states for later PCCA clustering was selected as 5. The results obtained are shown in Figure A3. If the solid line and dotted line in the picture are close to each other, it can be considered that the constructed Markov model has good Markov property. According to Figure A3, the constructed Markov model had good Markov property and could be used for subsequent research.

**Figure A4:**
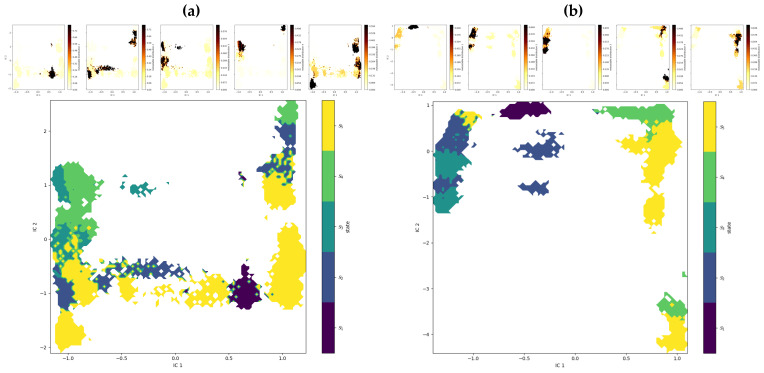

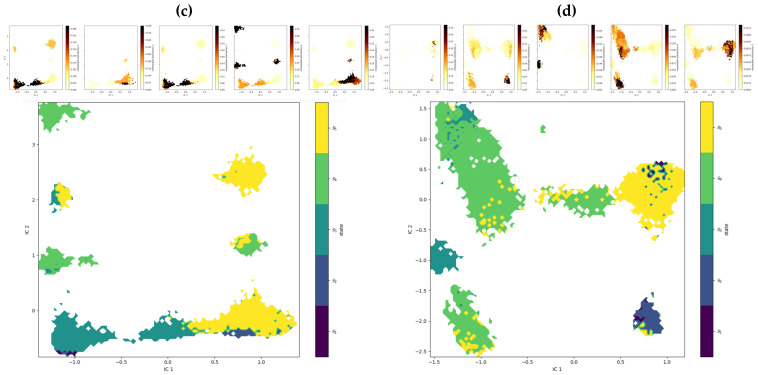
(**a**) The results of PCCA of Free-BRD4. (**b**) The results of PCCA of BRD4-ZL0590. (**c**) The results of PCCA of BRD4-MS436. (**d**) The results of PCCA of BRD4-MS436-ZL0590.

Next, PCCA clustering was performed to describe the molecular trajectory system as a macroscopic state. Five classes were selected for PCCA clustering, and the clustering results are shown in Figure A4. These two images represented the results of PCCA clustering. The above Figure shows the position of each class in the IC1 and IC2 dimensional after PCCA clustering. The following Figure shows the overall representation of molecular trajectory features in IC1 and IC2, and different colors are used to represent different PCCA classes. The SA in the clustering results represents the initial state of the conformation in the simulated trajectory, and the SB represents the final convergence state of the conformation in the simulated trajectory. S1, S2, and S3 are the states that occur during the trajectory process.

**Figure 8:**
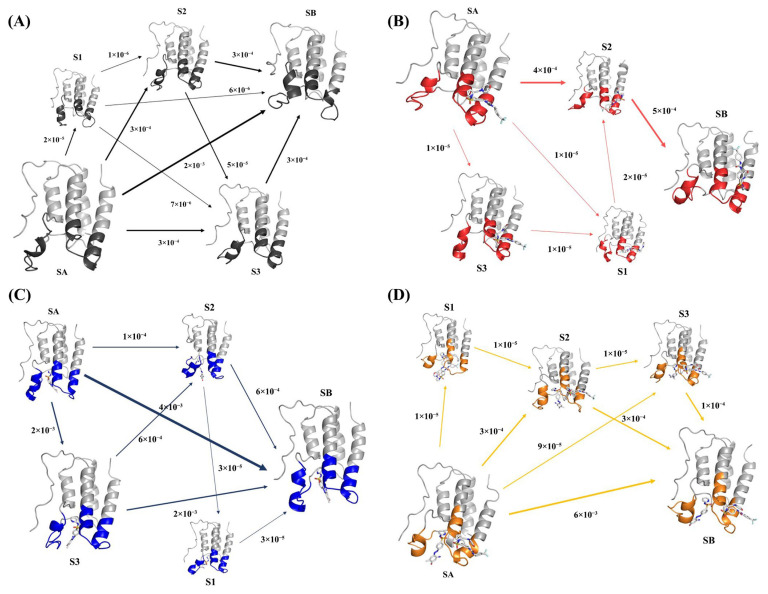
Flux analysis of Free-BRD4 (**A**), BRD4-ZL0590 (**B**), BRD4-MS436 (**C**), and BRD4-MS436-ZL0590 (**D**).

After obtaining various macroscopic states, flux analysis could be performed using these state results and the transition probability matrix of the Markov model. The flux analysis results are shown in Figure 8C (the blue marked area is the main focus of the studied α-helices region). By analyzing the flux and its proportion of each path, Table 1 could be obtained.

Observing this flux result, it can be seen that the most representative conformational transition pathways were SA→SB and SA→S3→SB, accounting for 59.17% and 29.59% of the total flux, respectively.

Therefore, based on the relationship between the secondary structural changes and flux analysis results, it can be seen that in the most representative SA→SB conformational transition, during the interaction between MS436 and BRD4, the degree of helix in the ZA loop region where BRD4 was originally in a helical state was reduced. In the conformational transition of SA→S3→SB, during the interaction between MS436 and BRD4, the ZA loop region of BRD4, which was originally in a helical state, first partially dissociated from the helix and then reformed into a helix, resulting in a decrease in overall helicity. The secondary structural transformation of the SA→S3→S2→SB and SA→S3→S2→S1→SB conformational transformation pathways was roughly similar to that of SA→S3→SB, both of which were the process of first unwinding the α-helices and then partially reforming it.

Similarly, dimensionality reduction, clustering analysis, Markov model construction, and flux analysis were performed on Free-BRD4, BRD4-ZL0590, and BRD4-MS436-ZL0590. The final results are shown in Figure 8A,B,D and Table 2, Table 3 and Table 4.

For BRD4-ZL0590, the most representative conformational transition pathway was SA→S2→SB, accounting for 95.24% of the total flux. Based on the relationship between the secondary structural changes and flux analysis results, it can be seen that in the most representative SA→S2→SB conformational transition, during the interaction between ZL0590 and BRD4, the degree of helix in the ZA loop region, which was originally irregularly curled, of BRD4 was increased. For BRD4-MS436-ZL0590, the most representative conformational transition pathway was SA→SB, accounting for 93.46% of the total flux. According to the relationship between the secondary structural changes and flux analysis results, it can be seen that in the most representative SA→SB conformational transition, the degree of helix in the ZA loop region of BRD4 increased during the interaction between MS436 and ZL0590 with BRD4.

### 2.5. Distance Analysis

**Figure 9:**
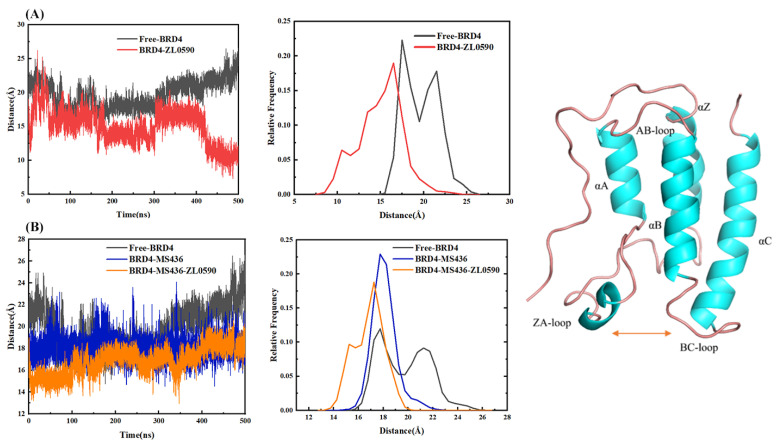
(**A**) The distance between BC loop and ZA loop of Free-BRD4 and BRD4-ZL0590. (**B)** The distance between BC loop and ZA loop of Free-BRD4, BRD4-MS436, and BRD4-MS436-ZL0590.

Since the active region exists between BC loop and ZA loop, in order to explore the conformational change at the active site in the binding process of the inhibitor, the distance between BC loop and ZA loop in the 500 ns molecular dynamics simulation was measured and analyzed. Figure 9 shows that the active pocket distance became smaller after the combination of allosteric inhibitors. The distance of the active pocket became larger after the binding of MS436, and the active pocket became smaller after the binding of allosteric inhibitor.

Figure 9A shows that the binding of allosteric inhibitors reduced the distance between ZA loop and BC loop, blocked the conformation at the active site, and inhibited the binding of substrates.

**Figure 10:**
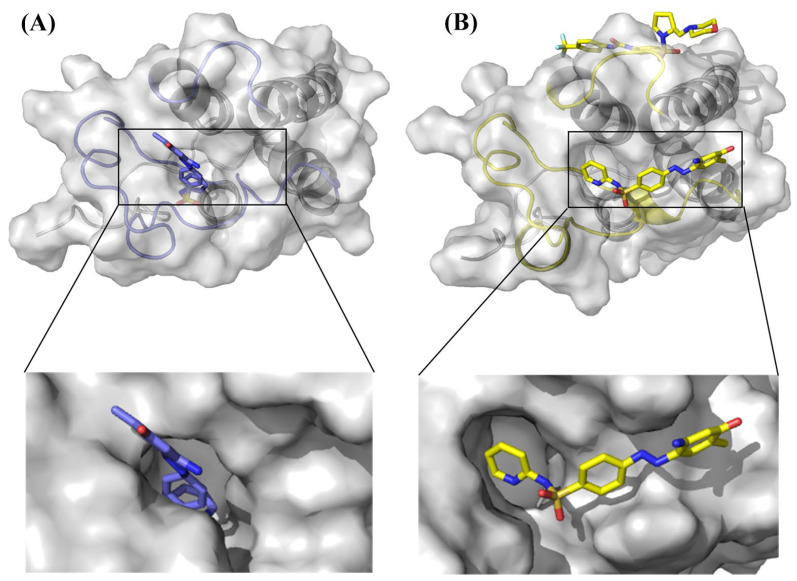
Schematic diagram of changes in active pockets. (**A**) MS436 in BRD4-MS436. (**B**) MS436 in BRD4-MS436-ZL0590.

After the combination of allosteric inhibitors, the distance between ZA loop and BC loop became smaller, and the orthotropic binding substrate that could normally penetrate deep into the pocket was floating on the edge of the active pocket but did not go further into the pocket as expected, indicating that allosteric regulation occurred. The binding of allosteric inhibitors prevented the normal binding of active sites (Figure 10).

### 2.6. Alanine Mutation Analysis

In this analysis, the residues around inhibitors (MS436 and ZL0590) were mutated into alanine, and after the active residues were replaced with alanine, the active groups on the side chain were removed and replaced with methyl groups, which had less influence on protein structure, to explore the influence of active residues on protein structural stability.

Table 5, Table 6 and Table 7 list the results. In Table 5, the mutation of ASP88 had the greatest impact on the BRD4-MS436 complex, and the mutation of other amino acids, such as PRO86 and GLN85, also had an impact on the energy change, indicating that ASP88 and PRO86 might play a key role in binding with BRD4 and MS436. Mutations for TYR137 in Table 6 had the greatest effect on the BRD4-ZL0590 complex, and mutations for TYR137 and TYR97 in Table 7 had the greatest effect on the BRD4-MS436-ZL0590 complex, suggesting that TYR137 might play a key role in binding to BRD4 and ZL0590.

## 3. Discussion

Bromodomains (BDs) protein modules are composed of approximately 110 amino acids and are responsible for identifying acetylated lysine (KAc) residues in proteins. This module is highly conserved in evolution and can function as a key member of epigenetics [23]. There are 61 types of BDs in the human body, which can be divided into 8 subtypes. Generally, according to the homology, quantity, and domain structure of BDs, BCPs can be divided into bromodomain, extra-terminal (BET), and non-BET families. BRD2, BRD3, BRD4, and testis-specific BRDT [24,25,26] all belong to the mammalian BET family. BRD4 is a member of the BET family and a multifunctional chromatin regulator. It plays a key role in DNA damage repair, cell state transition, congenital inflammation, and cell cycle progression, and can drive carcinogenic transformation [27,28]. 

Filippakopoulos et al. revealed the comprehensive structural characteristics of human BCPs and identified the Kac-specific recognition sites of these proteins [13]. Research shows that BDs have a relatively conservative fold, including four left-handed antiparallels α-helices (αZ, αA, αB, αC). They are connected by two hydrophobic loop zones (ZA and BC loops). These components form the pocket responsible for recognizing histone acetylation motifs. The eutectic structure data indicate that KAc is recognized by the central hydrophobic cavity [13].

Inhibiting BRD4 has been proven to be effective in treating malignant tumors with pathological activation of c-MYC [29,30]. BRD4 is widely recognized in cancer due to its role in super-enhancer (SE) tissue and oncogene expression regulation. The inhibition of BRD4 shortens the communication between SE and the target gene promoter, and then leads to the specific inhibition of oncogenes and cell death in cancer cells. Recent evidence suggests that the BRD4 protein has been identified as a maintainer of genomic stability. In fact, the role of BRD4 in controlling DNA damage checkpoint activation and repair, as well as telomere maintenance, has been proposed, providing new clues for the multiple functions of this protein [31].

Similarly, in acute myeloid leukemia, BRD4 has been shown to co-localize and synergistically interact with hematopoietic TFs (including PU.1, FLI1, ERG, C/EBPα, C/EBPβ, and MYB) combined with lysine acetyltransferase p300/CBP to support specific transcriptional circuits in this disease [32].

BRD4 has also been proven to be a necessary co-activator of the inflammatory transcription process driven by the combination of NF-κB with acetylated RelA, and a necessary co-activator of the diacetylated form of TWIST in triple negative breast cancer [33,34].

The proto-oncogene MYC regulates various cellular processes, including proliferation and metabolism. Maintaining MYC at a steady-state level is crucial for normal cell function, and its overexpression leads to many cancers. The stability of MYC is regulated through phosphorylation: the phosphorylation signal of Thr58 degrades, while the phosphorylation of Ser62 leads to its stability and functional activation. BRD4 is a transcription and epigenetics regulator with endogenous kinase and histone acetyltransferase (HAT) activity, which can activate the transcription of key proto-oncogenes, including BRD4 phosphorylating MYC at Thr58, leading to the ubiquitination and degradation of MYC, thus regulating MYC target genes. Importantly, the degradation of BRD4 rather than its inhibition leads to an increase in MYC protein levels. Conversely, MYC inhibits the HAT activity of BRD4, indicating that MYC regulates its own transcription by limiting chromatin remodeling mediated by BRD4. MYC stable kinase ERK1 directly or indirectly regulates MYC levels by inhibiting BRD4 kinase activity [35].

The interaction between BRD4 and p53 is mediated by two regions outside BD, or partially regulated by phosphorylation mediated by Casein kinase (CK2). CK2 phosphorylates BRD4 in the acidic region parallel to BD, enabling BRD4 to load on DNA and interact with p53 [31].

Among the DNA repair components that interact with BRD4 in the co-immunoprecipitation experiment, both works confirmed that p53 binding protein (53BP1) is the main binding partner of BRD4 in the regulation of DNA damage repair [36,37].

In addition, genes encoding BCPs often undergo chromosomal translocation and fusion [38]. Small molecule BD inhibitors directly bind to protein modules by mimicking the binding mode of KAc. In the past decade, the development of BCP inhibitors represented by (+)-JQ1 has made rapid progress [39,40,41].

Zhou’s team developed the BD1 selective inhibitor MS436 [42]. MS436 uses diazobenzene as its structural framework and can effectively bind to BRD4-BD1 (K_i_ = 30–50 nM), with a selectivity of approximately ten times that of BD2. The Department of Pharmacology of the University of Texas Medical Division (UTMB), based on the lead compound ZL0454, carried out further structural optimization to obtain ZL0590, which was bound to a new allosteric site [18] instead of the classic acetylated lysine KAc binding pocket. This allosteric site is located at α-helices B and C, back to back with classic KAc pockets [18]. By oral administration, compound ZL0590 shows inhibition of poly (I: C) induced airway inflammation, weakening inflammatory secretion, and blocking acute airway inflammation.

The results of this study show that, from the perspective of the entire system, the stability of BRD4-ZL0590 was lower than that of BRD4-MS436, which might be due to the hydrogen bonding between ZL0590 and BRD4 being less than that of MS436 (as shown in Figure 1). By comparing the detailed interactions reported in the literature between MS436 [42], ZL0590 [18], and BRD4, it can also be seen that there was more interaction between BRD4 and MS436 than between ZL0590. From Figure 1, it can be seen that compared to BRD4-MS436-ZL0590, the overall interaction between inhibitors and proteins was more pronounced, while the interaction between MS436 and proteins was less pronounced, resulting in reduced stability. The RMSF diagram also shows that the flexibility of BRD4-MS436 was greater than that of BRD4-MS436-ZL0590 at residues 90–110. The residues 90–110 in Free-BRD4 exhibited higher flexibility, while in BRD4-MS436-ZL0590, their value of RMSF was reduced compared to BRD4-MS436. The results indicate that binding allosteric inhibitors might enhance the interaction between BRD4 and MS436 between residues 90 and 110. Referring to the whole molecular dynamics simulation process, it could be inferred that in BRD4-MS436-ZL0590, because MS436 failed to penetrate into the active site, the binding between MS436 and protein floated at the edge of the active pocket, and the interaction was reduced. However, the hydrophobic loops ZA loop and BC loop did not fully interact with MS436, resulting in an increase in the hydrophobicity of BRD4-MS436-ZL0590.

The normal inhibitor is a competitive inhibitor, and the normal structure site is the active site. After the allosteric inhibitor was combined, the distance between ZA loop and BC loop became smaller. The normal-binding inhibitor that could normally penetrate into the inside of the pocket floated at the edge of the active pocket and did not continue to penetrate as expected, indicating that allosteric regulation had occurred, and the binding of allosteric inhibitors had hindered the normal binding of the active site (Figure 10). The impact of allosteric inhibitors on active sites was indeed evident, as Van der Waals interactions near the active site were significantly reduced, indicating that the addition of allosteric inhibitors affected the interaction of the active site and thus affect the stability of binding.

The results of the Markov model showed that the conformation of the Free-BRD4 system as a control group did not show significant changes. The most representative conformational transition pathway (SA→S2→SB) of the BRD4-ZL0590 system showed an increase in the degree of helix in the ZA loop region; the most representative conformational transition pathway (SA→SB) of the BRD4-MS436 system showed a decrease in the degree of helix in the ZA loop region; and the most representative conformational transition pathway (SA→SB) of the BRD4-MS436-ZL0590 system showed an increase in the degree of helix in the ZA loop region. The increase in protein helicity is beneficial for the stability of the complex structure. The results show that the binding of allosteric inhibitor ZL0590 had a high probability (95.24%) of causing BRD4 to form an S2 microstate. At this time, the degree of helix in the ZA loop region increased, and the helix further twisted, ultimately forming an SB microstate. The combination of MS436 had a probability of about 60% that it would cause BRD4 to directly transition from the initial state SA to the final state SB, reducing the degree of helicity; there was a 30% probability that BRD4 would form an S3 microstate, at which point the ZA loop region would undergo a process of first partially decomposing the helix and then reforming the helix. The combination of MS436 and ZL0590 also had a high probability (93.46%) of leading to a direct conformational transition from SA to SB, resulting in an increase in the degree of helicity. The results of Markov model flux analysis explained the impact of the binding of allosteric inhibitor ZL0590 on the conformational changes in Free-BRD4 and BRD4-MS436.

Molecular dynamics simulation can capture the position and motion of each atom at each time point [43], and Markov models can analyze the conformational change paths during the binding process of small molecules [44]. Using molecular dynamics simulation combined with Markov models to analyze the interaction relationship and dynamic binding mechanism between BRD4 protein and small molecule inhibitors is expected to provide some clues for subsequent experimental research and small molecule drug development.

## 4. Materials and Methods

### 4.1. System Preparation

In order to explore the mechanism of allosteric sites, we designed four different systems, which were divided into two groups for analysis, namely, Free-BRD4, BRD4-ZL0590, and Free-BRD4, BRD4-MS436, BRd4-MS466-ZL0590. The 3D structures of BRD4-MS436 (PDB code: 4NUD) [42] and BRD4-ZL0590 (PDB code: 6u0d) [18] were obtained from the protein database (www.rcsb.org, accessed on 2 August 2022). The water and ligand in the protein were removed by Discovery Studio [45], and Free empty protein Free-BRD4 was obtained.

According to the crystal structure downloaded from the PDB database, we could accurately obtain the binding site of the active site and allosteric site. The structure that needed docking was BRD4-MS436-ZL0590. We used Autodock Vina [46] for molecular docking. Vina eliminates the need to select atomic types and predictive grid diagrams for ligands and receptors, significantly improves the average accuracy of combined model predictions, and speeds up the search by using simpler scoring functions. Thus, we obtained the complex BRD4-MS436-ZL0590, which was combined with the inhibitor MS436 and ZL0590.

### 4.2. Molecular Dynamics Simulations

In this experiment, Amber 16 software(version number: Amber 16) [47,48] was used to perform 500 ns molecular dynamics simulation on four systems of Free-BRD4, BRD4-ZL0590 allosteric inhibitor complex and Free-BRD4, BRD4-MS436 normal inhibitor complex, and BRD4-MS436-ZL0590 double-site binding complex. Amber FF99SB force field [49,50] was applied to BRD4. The TIP3P model [51,52] was adopted, and periodic boundary conditions were applied to the reaction system in the simulation process to prevent edge effect. The distance between the solute surface and the box was set to 12 Å. Since the charge in the initial reaction system was not zero, Na+ should be added to the system in the initial stage of reaction simulation.

After the construction of the system, the energy of the four systems was minimized in order to eliminate atomic collisions in the initial structure. The whole process was divided into two parts: the steepest descent method and conjugate gradient method. After the energy was minimized, the initial structure of the system was stable, and the simulated reaction temperature was increased from 0 K to 300 K with a reaction time of 50 ps. After heating, the simulation system then treated the density equilibrium with a reaction time of 50 ps. Finally, the simulation system was balanced at constant pressure under NPT ensemble, and the constant pressure balance time was 500 ps at 300 K. Constant pressure equilibrium was the last step in system equilibrium. After the thermodynamic parameters were stabilized, molecular dynamics of the four simulation systems were simulated at 500 ns. Each system had a storage interval of 10 ps/interval and a total recording structure of 50,000 frames. These data were preserved for further study and analysis.

### 4.3. Trajectory Analysis

Trajectory analysis included RMSD, R_g_, SASA, RMSF, and distance analysis, calculated using the Amber16 cpptraj module [53]. B-factor data were calculated using the cpptraj module, processed using Python scripts, visualized using Pymol [54]. The Prody arrow trends were analyzed using VMD [55], and DCCM-PCA was calculated using R-STUDIO [56].

### 4.4. Markov Model

Markov models can cluster repetitive states in molecular trajectories that have very close conformations into a class called a microstate. Each microstate corresponds to a state in the Markov model state space, and there is a transition probability between each state, forming a transition probability matrix. By using the transfer probability matrix and flux analysis methods, the dynamic relationships between various microstates can be analyzed [57].

#### 4.4.1. TICA Dimensionality Reduction Method

Time-lagged independent component analysis (TICA) is a commonly used dimensionality reduction method in Markov model construction. The Markov model uses high-dimensional data to describe trajectory information, and extracts information from 10 or more repeated trajectories using the input molecular trajectories. The extracted trajectory information is usually high-dimensional data, which are difficult to directly use. If used directly, they will consume a lot of computing resources and time, which is very unfavorable. Therefore, the TICA dimensionality reduction method plays an important role.

The TICA algorithm is one of the ICA algorithms, known as independent component analysis. The ICA algorithm does not analyze the principal components of the data during dimensionality reduction but considers all components equally important. TICA is an algorithm used for dimensionality reduction of high-dimensional data, which can maximize the autocorrelation of transformation coordinates and is particularly suitable for molecular dynamics inputs. TICA can find the coordinates of the maximum autocorrelation at a given lag time. This feature enables TICA to effectively extract slow order parameters from molecular dynamics data, making it an excellent choice for processing molecular simulation data before k-means clustering [58].

Since the constant temperature molecular dynamics simulation is a Markov process, when the molecular dynamics simulation data are used as the input data of TICA, TICA actually finds the characteristic function and approximate value of the characteristic value of the underlying Markov operator, and then the TICA conversion value can be estimated based on the input data according to the approximate value. When input data are given as a parameter, estimation will be performed immediately, and this result can be used to obtain eigenvalues, eigenvectors, or project input data onto the slowest TICA component.

#### 4.4.2. K-Means Clustering Algorithm

The k-means clustering algorithm is a common algorithm in machine learning, and is one of the non-supervised learning methods. The core of this algorithm is to partition an unlabeled dataset without prior knowledge into k parts by optimizing the evaluation function. The k-means algorithm is a non-convex algorithm; therefore, it has a local optimal solution. In order to avoid the occurrence of local optimal solutions, the commonly used method is to attempt random initialization for some times, with the expected optimal solution being the global optimal solution. Therefore, in this experiment, the k-means clustering step was repeated 10 times for each k-value to calculate the score. At the same time, it is difficult to determine the super-parameter k in the k-means algorithm, which can be determined using the elbow rule. However, in reality, the elbow rule is not effective in many cases. Therefore, this experiment uses the idea of the elbow rule to simplify the calculation steps, and only conducts a grid search for more reasonable k-values. By scoring the clustering results of this k-value with VAMP, we can compare which k-value is the most appropriate [59,60].

In the Markov model, the main reason for using k-means clustering is to cluster the conformational states in the molecular simulation trajectories through clustering. Each clustered class is defined as a microstate in the Markov model, which results in a discrete trajectory of microstates. Afterwards, the Markov model can calculate the transition probability between microstates based on the discrete trajectory of microstates, and obtain the transition probability matrix.

#### 4.4.3. Determination of Lag Time

Lag time is an extremely important parameter in the Markov model that calculates the transition probability matrix through discrete trajectories, which directly determines the value of the transition probability matrix. The physical meaning of lag time is the time used for each jump between discrete trajectories.

In practical calculations, after obtaining the discrete trajectories of microstates, a lag time jump is performed along these discrete trajectories, and the jump information generated during the process is recorded in the counting matrix, which is then converted into a transition probability matrix.

Markov time is the minimum lag time that can provide Markov behavior, which refers to memoryless behavior. The method for making the lag time reach Markov time is to evaluate the loss of lag time dependence on the implied relaxation timescale in the system [61,62].

Lag time has a significant impact on Markov models. If lag time is too small, there is a significant difference between the eigenvector error and spectral error of the Markov model. If the lag time is too large, numerical errors can have a significant impact.

In order to test whether the selection of lag time is appropriate, the Chapman–Kolmogorov test is often used for testing [63]. This method detects by comparing the left and right sides of the Chapman–Kolmogorov equation.

$$P\left(k\tau \right)=P^{k}\left(\tau \right)$$

Among them, P(τ) is the transition probability matrix determined by lag time τ, while kτ is represented as a multiple of τ. The lag time τ is performed with multiple amplifications and then a Markov model is constructed to obtain the transition probability matrix. Comparing this result with the transition probability matrix of the k-power calculated by the original τ, if the left and right sides of the equation are equal or very close, it indicates the determined lag time τ can make the Markov model become memoryless, and usually the choice of lag time is considered appropriate when the two sides of the equation are close to 95% [64].

#### 4.4.4. PCCA+ Clustering Algorithm

The PCCA+ clustering algorithm is known as Perron cluster analysis. PCCA is not used to handle conventional clustering problems, but specifically for the processing of microstates in Markov models.

Due to the large order of magnitude of the microstates obtained through k-means algorithm clustering, such a large quantity is difficult to use for intuitive analysis, and is also extremely large for subsequent flux analysis processing. Therefore, using PCCA+ clustering algorithm to divide the trajectory conformation into multiple macroscopic states for analysis is a good method [65].

Usually, a molecular dynamics simulation trajectory only contains up to 10 conformational states. The k-means clustering results usually contain more than 100 classes. Therefore, at this point, the PCCA+ clustering algorithm is used to cluster multiple microstates into several metastable states with longer durations using the dynamic information provided by the completed MSM model. After such processing, it can facilitate subsequent flux analysis [66].

#### 4.4.5. Flux Analysis

If the molecular simulation trajectories have been clustered into macroscopic metastable states, flux analysis can be performed. In fact, after the transfer probability matrix is obtained using the Markov model, we can use this result to obtain the thermodynamic and dynamic information of the trajectory. However, because this model has a high complexity, it may be necessary to use a more coarse-grained model to provide the same quantitative information in a more compact way, so it can be used for flux analysis [67].

The purpose of flux analysis is to obtain transfer trajectories from the microstate space constructed by the MSM model, so probability distribution matrices and flux are required.

The central idea is to calculate the forward probability *q_i_*^+^ and backward probability *q_i_*^−^ for each microstate when it is stable. The calculation methods for *q_i_*^+^ and *q_i_*^−^ are as follows [68]:

$$q_{i}^{+}=\underset{j\in B}{\sum }T_{ij}+\underset{j\in \bar{A\cup B}}{\sum }T_{ij}q_{j}^{+}$$

$$q_{i}^{-}=1-q_{i}^{+}$$

Among them, *T_ij_* is the transition probability between two given microstates, provided by the transition probability matrix of the Markov model, and *A* and *B* are the two endpoint microstates, respectively [62].

After calculating *q_i_*^+^ and *q_i_*^−^, the effective flux *f_ij_* of microstate *i* transition to microstate *j* can be calculated based on this. The calculation formula is as follows, where *p_i_* is the Boltzmann probability of state *i*.

$$f_{ij}=\rho_{i}q_{i}^{-}T_{ij}q_{j}^{+}$$

Therefore, the net flux from microstate *i* to microstate *j* can be calculated using two effective fluxes, *f_ij_* and *f_ji_*:

$$f_{ij}^{+}=max\left(f_{ij}-f_{ji}\right)$$

According to this method, the net flux between any two microstates can be calculated.

For multiple metastable states in molecular dynamics simulation, it is only necessary to clarify the initial metastable state A and final metastable state B of the trajectory, and then determine the intermediate states between the two endpoints of AB based on the transfer probability matrix and flux between the metastable states.

Due to the relationship between transfer probabilities, intermediate states are not unique, but there may be multiple situations. Each state situation will determine an occurrence probability based on the flux on its path. The calculation method for path probability is as follows:

$$P_{i}^{A\to B}=\frac{f_{i}^{A\to B}}{\sum_{j}f_{j}^{A\to B}}$$

After calculating the probabilities of each path, the conformational changes of each path and the probability of its occurrence can be used for path analysis.

If you want to combine the cluster size of each metastable state, which is also known as the frequency of metastable state occurrence, with the flux probability, you can assign an allocation coefficient to each metastable state based on the number of times this metastable state is observed in the path, and calculate the total probability by weighting it based on the probability of the path and route. Ultimately, the path order with the highest probability can be obtained.

### 4.5. Alanine Scanning

Using Discovery Studio, alanine scans were performed near the active and allosteric sites of BRD4-MS436, BRD4-ZL0590, and BRD4-MS436-ZL0590 to explore key residues that may play a role in inhibitor binding.

## 5. Conclusions

In this study, 500 ns molecular dynamics simulation was used to study four systems: Free-BRD4, BRD4-ZL0590, BRD4-MS436, and BRD4-MS436-ZL0590. The results showed that in the simulation process of 500 ns MD, the secondary structure conformation of residues M90-A110 changed after combining with the normal inhibitor MS436, while the allosteric inhibitor ZL0590 was combined and the α-helices caused by MS436 unraveled. The binding of allosteric inhibitors reduced the distance between ZA loop and BC loop, blocked the conformation at the active site, and inhibited the binding of the substrate. After the binding of allosteric inhibitors, the distance between ZA loop and BC loop became smaller, and the normal-binding substrate that could normally penetrate into the interior of the pocket was floating on the edge of the active pocket but did not penetrate further as expected, indicating that allosteric regulation occurred, and the binding of allosteric inhibitors hindered the normal binding of the normal structure site. Our results may provide clues for the design of new cancer target inhibitors.

## Figures and Tables

**Table 1:** The flux analysis data of BRD4-MS436.

Pathways	Path Flux (s^−1^)	Percentage of Total Coarse Flux (%)
SA→SB	4.00 × 10^−3^	59.17
SA→S3→SB	2.00 × 10^−3^	29.59
SA→S3→S2→SB	6.00 × 10^−4^	8.88
SA→S2→SB	1.00 × 10^−4^	1.48
SA→S3→S2→S1→SB	3.00 × 10^−5^	0.44
SA→S2→S1→SB	3.00 × 10^−5^	0.44
Total	6.76 × 10^−3^	100

**Table 2:** The flux analysis data of Free-BRD4.

Pathways	Path Flux (s^−1^)	Percentage of Total Coarse Flux (%)
SA→SB	2.00 × 10^−3^	75.19
SA→S2→SB	3.00 × 10^−4^	11.28
SA→S3→SB	3.00 × 10^−4^	11.28
SA→S2→S3→SB	5.00 × 10^−5^	1.88
SA→S1→S3→SB	7.00 × 10^−6^	0.26
SA→S1→SB	6.00 × 10^−6^	0.23
SA→S1→S2→SB	1.00 × 10^−6^	0.04
SA→S1→S2→S3→SB	1.00 × 10^−6^	0.04
Total	2.66 × 10^−3^	100

**Table 3:** The flux analysis data of BRD4-ZL0590.

Pathways	Path Flux (s^−1^)	Percentage of Total Coarse Flux (%)
SA→S2→SB	4.00 × 10^−4^	95.24
SA→S1→S2→SB	1.00 × 10^−5^	2.38
SA→S3→S1→S2→SB	1.00 × 10^−5^	2.38
Total	4.20 × 10^−4^	100

**Table 4:** The flux analysis data of BRD4-MS436-ZL0590.

Pathways	Path Flux (s^−1^)	Percentage of Total Coarse Flux (%)
SA→SB	6.00 × 10^−3^	93.46
SA→S2→SB	3.00 × 10^−4^	4.67
SA→S3→SB	9.00 × 10^−5^	1.40
SA→S2→S3→SB	1.00 × 10^−5^	0.16
SA→S1→S2→SB	1.00 × 10^−5^	0.16
SA→S1→S2→S3→SB	1.00 × 10^−5^	0.16
Total	6.42 × 10^−3^	100

**Table 5:** Alanine mutation of BRD4-MS436.

Mutation	Mutation Energy (kcal/mol)	Effect
W81A	−0.59	STABILIZING
P82A	−0.54	STABILIZING
F83A	−0.05	NEUTRAL
Q85A	−0.85	STABILIZING
P86A	−1.05	STABILIZING
V87A	0.01	NEUTRAL
D88A	−1.11	STABILIZING
K91A	−0.63	STABILIZING
L92A	0.12	NEUTRAL
L94A	−0.21	NEUTRAL
Y97A	0.31	NEUTRAL
M105A	−0.56	STABILIZING
M132A	−0.06	NEUTRAL
N135A	−0.33	NEUTRAL
C136A	−0.29	NEUTRAL
Y139A	0.12	NEUTRAL
N140A	0.18	NEUTRAL
I146A	0.44	NEUTRAL

**Table 6:** Alanine mutation of BRD4-ZL0590.

Mutation	Mutation Energy (kcal/mol)	Effect
T134A	0.01	NEUTRAL
T137A	1.1	DESTABILIZING
I138A	0.63	DESTABILIZING
N140A	−0.04	NEUTRAL
K141A	0.03	NEUTRAL
P142A	0.22	NEUTRAL
G143A	−0.2	NEUTRAL
V147A	0.57	DESTABILIZING
L148A	0.1	NEUTRAL
E151A	0.1	NEUTRAL

**Table 7:** Alanine mutation of BRD4-MS436-ZL0590.

Mutation	Mutation Energy (kcal/mol)	Effect
W81A	0.45	NEUTRAL
P82A	0.15	NEUTRAL
F83A	0.44	NEUTRAL
Q85A	−0.64	STABILIZING
P86A	−0.03	NEUTRAL
V87A	0.57	DESTABILIZING
L92A	0.81	DESTABILIZING
L94A	0.46	NEUTRAL
Y97A	0.94	DESTABILIZING
M102A	0.01	NEUTRAL
D106A	0	NEUTRAL
M132A	0.07	NEUTRAL
T134A	0.07	NEUTRAL
N135A	−0.01	NEUTRAL
C136A	0.07	NEUTRAL
Y137A	0.94	DESTABILIZING
I138A	0.81	DESTABILIZING
Y139A	0.35	NEUTRAL
N140A	0.53	DESTABILIZING
K141A	0.06	NEUTRAL
P142A	0.54	DESTABILIZING
G143A	−0.2	NEUTRAL
I146A	0.29	NEUTRAL
V147A	0.08	NEUTRAL
L148A	0.19	NEUTRAL
A150A	0	NEUTRAL
E151A	−0.03	NEUTRAL

## Data Availability

Not applicable.
